# Editorial: TNFR Superfamily Oligomerization and Signaling

**DOI:** 10.3389/fcell.2021.682472

**Published:** 2021-04-20

**Authors:** Olivier Micheau, Marta Rizzi, Cristian R. Smulski

**Affiliations:** ^1^INSERM, LNC, UMR 1231, Dijon, France; ^2^Université de Bourgogne Franche-Comté, Dijon, France; ^3^Department of Rheumatology and Clinical Immunology, Faculty of Medicine, University Medical Center Freiburg, University of Freiburg, Freiburg, Germany; ^4^Medical Physics Department, Bariloche Atomic Centre Comisión Nacional de Energía Atómica (CNEA) and Consejo Nacional de Investigaciones Científicas y Técnicas (CONICET), San Carlos de Bariloche, Argentina

**Keywords:** TNF/TNFR superfamily, oligomerization, signaling, therapeutic targets, specific targeting

## Introduction

The TNF/TNFR superfamily comprises 19 ligands and 30 receptors, all representing therapeutically relevant targets in a wide range of human diseases (Micheau, 2017; Yi et al., 2018). TNF family ligands are type 2 membrane bound proteins with a common structural motif that mediates ligand trimerization: the TNF homology domain (Bodmer et al., 2002). Each trimer subunit binds to one TNF receptor (TNFR) subunit, inducing receptor oligomerization that represents the minimal active unit in most members of the family. The outcome of signaling following ligand binding results from the interplay of different factors: ligand architecture, assembly of receptor units in the appropriate location, posttranslational modifications and transmembrane helix associations. The “TNFR Superfamily Oligomerization and Signaling” Research Topic covers many of these features and provides new insights into complex regulatory mechanisms.

## TNFR Targeting And Signaling Modulation

TNF-TNFR1 constitutes one of the most studied ligand-receptor pairs of the family. Signaling outcome ranges from NF-kB and MAPK activation to apoptosis or necroptosis (Ting and Bertrand, 2016). To achieve this, a number of events, including phosphorylation and ubiquitination are triggered upon ligand binding. These events are tightly regulated by a plethora of molecules that dictates the signaling outcome. Disruption of these events can lead to severe inflammatory diseases as reviewed by Webster and Vucic Notably, TNF can bind to a second receptor of the family, TNFR2, whose immune function differs from TNFR1. Indeed, several TNF family ligands can bind to more than one receptor. Because of this, ligand-blocking therapies are likely to affect a handful set of ligand-receptor pairs with unwanted effects. To overcome this problem, selective targeting of TNFR1 and TNFR2 has been developed showing a great therapeutic potential in several diseases as reviewed by  Fischer et al. In the context of selective targeting of TNFR1 and 2, Wei et al. proposed a model in which the engineered protein “Atsttrin” (a derivative of progranulin) promotes cartilage repair primarily through TNFR2-Akt pathway, despite being able to bind and signal through both receptors.

Another example of a ligand binding to several receptors is TRAIL. This ligand can bind to two decoy receptors (DcR1 and DcR2) and to two functional receptors (DR4 and DR5) (LeBlanc and Ashkenazi, 2003). Binding to DR4 and DR5 triggers apoptosis in cancer cells (French and Tschopp, 1999). However, resistance to TRAIL induced apoptosis has been described in several tumor cells (Deng and Shah, 2020). Setroikrom et al. have described a novel mechanism of resistance in colon cancer cells through selective segregation of DR5 into extracellular vesicles (EVs). Indeed, membrane bound receptors through EVs may not only modulate signaling pathways associated with TNF/TNFR superfamily members, but are likely to mediate communication between cells in complex systems.

## Oligomerization Of Ligands And Receptors

Amongst the TNF family ligands, the B cell activating factor (BAFF) has a unique feature that allows assembly of 20 adjacent trimers in a virus-like capsid called BAFF 60-mer, resulting in stronger activation of its receptors, *in vitro* (Cachero et al., 2006; Vigolo et al., 2018). However, it is unclear which is the physiological form of soluble BAFF in humans. Eslami et al. investigated the presence of highly oligomeric forms of BAFF in different human fluids, and detected high molecular weight forms of BAFF only in cord blood. This BAFF displayed some but not all properties of BAFF 60-mer. Moreover, an activity that dissociates BAFF 60-mer into trimers was identified which could be related to the exclusive presence of BAFF 3-mer in adult human serum and cerebrospinal fluid.

Ligand-induced receptor oligomerization is a critical step for signal transduction. Noteworthy, not all receptors of the family respond similarly to soluble or membrane-bound ligands. This phenomenon seems to be related to the oligomeric threshold intrinsic to each signaling pathway.  Kucka and Wajant have reviewed the most relevant aspects of receptor oligomerization for TNFRs signaling, including clustering of free and bound TNFRs, receptor oligomerization requirement for specific signaling pathways, and how this knowledge contributes for the rational design and development of TNFR agonists that target specific members of the family. Moreover, Levoin et al. have described, in a comprehensive manner, how CD95 sub-domains and their post-translational modifications contribute to receptor aggregation and cell signaling, upon binding to different ligand forms. One emerging property of TNFRs is the ability of their transmembrane domains to mediate ligand independent associations. In the current topic Zhao et al. have solved the trimeric structure of transmembrane domain of TNFR1. Comparison of this structure with that of Fas and DR5 revealed similarities such as trimerization, but also significant structural divergences, undserscoring the importance of a systematic investigation of other TNFR family members. In line with this conclusion, Sica and Smulski have analyzed and compared the assembly of the previously solved transmembrane regions of p75NTR, Fas, and DR5 using coarse grained molecular dynamic simulations. This tool has proven useful for unbiassed prediction of oligomerization levels, residues involved in interactions, and impact of disease-associated mutations in this region.

Overall, the current Research Topic has covered important landmarks of the macromolecular complexes and signaling pathways engaged by TNF/TNFR family members. Although other features must be understood for proper selective therapeutic intervention, including spatial localization and function of the receptors (Staniek et al., 2019), their post-translational modifications, that may directly affect their signal transduction capabilities (Wagner et al., 2007; Dufour et al., 2017), receptor shedding and decoy activities (Hoffmann et al., 2015; Laurent et al., 2015; Smulski et al., 2017b), and potential interactions between members of the family (Smulski et al., 2017a). Understanding the complex function and signaling interplay can be exploited to design effective treatments, as recently shown in experimental melanoma (Bertrand et al., 2017; Montfort et al., 2019). Inhibition of TNF-induced TNFR1 signaling is sufficient to overcome resistance to immune checkpoint inhibitors, restoring the anti-tumoral immune response. The ongoing clinical trials translating this finding are promising (Montfort et al., 2021), showing high response rates in advanced and/or metastatic melanoma patients. Finally, despite the fact that most family members have been known for more than two decades, including TNF and TNFR1, new therapeutic opportunities may emerge as a result of our better understanding of TNF/TNFR family members.

## Author Contributions

All authors listed have made a substantial, direct and intellectual contribution to the work, and approved it for publication.

## Conflict of Interest

The authors declare that the research was conducted in the absence of any commercial or financial relationships that could be construed as a potential conflict of interest.
